# Long non-coding RNA JPX promotes endometrial carcinoma progression via janus kinase 2/signal transducer and activator of transcription 3

**DOI:** 10.3389/fonc.2024.1340050

**Published:** 2024-05-09

**Authors:** Hanzhen Xiong, Wei Zhang, Mingyu Xie, Ruichao Chen, Hui Chen, Qiongyan Lin

**Affiliations:** ^1^ Department of Pathology, Guangdong Provincial Key Laboratory of Major Obstetric Disease, Guangdong Provincial Clinical Research Center for Obstetrics and Gynecology, Third Affiliated Hospital of Guangzhou Medical University, Guangzhou, Guangdong, China; ^2^ Department of Pathology, Central People’s Hospital of Zhanjiang, Zhanjiang, Guangdong, China; ^3^ Department of Obstetrics and Gynecology, Guangdong Provincial Key Laboratory of Major Obstetric Diseases, Guangdong Provincial Clinical Research Center for Obstetrics and Gynecology, Guangdong-Hong Kong-Macao Greater Bay Area Higher Education Joint Laboratory of Maternal-Fetal Medicine, The Third Affiliated Hospital of Guangzhou Medical University, Guangzhou, Guangdong, China; ^4^ Department of Gynecologic Oncology Research Office, Guangdong Provincial Key Laboratory of Major Obstetric Diseases, Guangdong Provincial Clinical Research Center for Obstetrics and Gynecology, Guangdong-Hong Kong-Macao Greater Bay Area Higher Education Joint Laboratory of Maternal-Fetal Medicine, The Third Affiliated Hospital of Guangzhou Medical University, Guangzhou, Guangdong, China

**Keywords:** Jpx, MiR-140-3p, PIK3CA, JAK2/STAT3, endometrial carcinoma

## Abstract

**Introduction:**

Although LncRNA JPX has been linked to a number of malignancies, it is yet unknown how it relates to endometrial carcinoma (EC). Investigating the expression, functional activities, and underlying molecular processes of lncRNA JPX in EC was the goal of this work.

**Methods:**

RT-qPCR was used to examine the differences in lncRNA/microRNA (miRNA, miR)/mRNA expression between normal cervical and EC tissues or cells. Cell Counting Kit-8, flow cytometry, and transwell were used to evaluate the association between lncRNA JPX/miR-140-3p/phosphoinositide-3-kinase catalytic subunit α (PIK3CA) in Ishikawa and JEC cell lines. The impact of JPX on the downstream janus kinase (JAK)2/signal transducer and activator of transcription (STAT)3 signaling pathway was investigated using Western blot analysis.

**Results:**

When comparing EC tissues to nearby normal tissues, JPX expression is markedly increased in EC tissues, with greater expression in advanced-stage EC. Furthermore, compared to normal epithelial cells, EC cell lines have higher levels of JPX expression. In Ishikawa and JEC endometrial cancer cell lines, we used siRNA-mediated suppression of JPX to find lower cell viability, increased apoptosis, cell cycle arrest, and reduced migration and invasion. We next verified that miR-140-3p binds to downstream target cells to impede the transcription and translation of PIK3CA, which in turn prevents the growth of Ishikawa and JEC cells. JPX functions as a ceRNA to adsorb miR-140-3p. This procedure required controlling JAK2/STAT3, a downstream signal.

**Conclusion:**

JPX enhances the development of Ishikawa and JEC cells and activates downstream JAK2/STAT3 signal transduction via the miR-140-3p/PIK3CA axis, offering a possible therapeutic target for the treatment of EC.

## 1 Introduction

Endometrial carcinoma (EC) is a prevalent malignancy of the female reproductive system and poses a significant health burden worldwide (1). Although early diagnosis can achieve a good prognosis (2), the mortality rate of advanced EC remains high (3). Therefore, understanding the molecular mechanisms underlying EC pathogenesis and identifying novel therapeutic targets are of utmost importance.

Transcripts that are longer than 200 nucleotides but do not encode proteins are known as long non-coding RNAs, or lncRNAs. The development of EC is intimately linked to lncRNA dysregulation (4, 5). Among them, lncRNA JPX, a known oncogene, promotes the progression of gastric cancer (6), cervical cancer (7) and non-small cell lung cancer (8). *In vitro* studies of the molecular processes of EC cells often make use of the Ishikawa and JEC cell lines (9, 10). However, nothing is known about JPX’s involvement in the emergence and advancement of EC. Furthermore, lncRNAs bind to microRNAs (miRNAs) like natural sponges and function as competitive endogenous RNAs (ceRNAs) (11).

In order to regulate post-transcriptional gene expression, miRNA primarily bind to the 3’ untranslated region (3’ UTR) of target mRNAs. Treatment for EC requires regulation of miRNAs (12). Of them, reduced expression of miR-140-3p is associated with an increased risk of developing and a poor prognosis for a number of malignancies, such as cervical (13) and colon cancers (14). Notably, there hasn’t been any information released on miR-140-3p’s possible impact on EC. By attaching to messenger RNA (mRNA), miRNAs may influence the transcription and translation of mRNA.

The proto-oncogene phosphoinositide-3-kinase (PI3K) catalytic subunit α gene (*PIK3CA*) located on chromosome 3 promotes the progression and poor prognosis of various cancers, such as breast (15) and rectal cancers (16). Studies have confirmed that PIK3CA is an important factor in the growth of EC (17, 18). By inhibiting the expression or mutation of *PIK3CA*, the development of EC can be slowed down.

The primary signal transduction route of many cytokines is the Janus kinase (JAK)-signal transducer and activator of transcription (STAT) signaling pathway (19). Research has shown that PIK3CA may control the JAK-STAT downstream pathway to influence the development of rectal cancer (20) and renal cell carcinoma (21). A crucial component of the JAK-STAT signaling cascade, JAK2-STAT3 is intimately linked to several cell processes, including migration, cycle, apoptosis, and cancer cell survival (22–24). However, JPX/miR-140-3p/PIK3CA axis in EC, as well as their influence on the JAK2/STAT3 pathway, remains unclear.

In this study, we examined the function of lncRNA JPX in the initiation and evolution of EC, its association with miR-140-3p, and its influence on the level of PIK3CA and the JAK2/STAT3 signaling cascade. Our research sheds light on the molecular processes behind EC and might lead to the creation of fresh treatment approaches for the condition’s management.

## 2 Materials and methods

### 2.1 Study subjects

Between July 2020 and October 2022, a total of 32 pairs of CE and adjacent tissues were obtained from 32 female patients with EC (age range: 42-68) at the Third Affiliated Hospital of Guangzhou Medical University. Informed consent forms were signed by all patients or their families, and paracancerous tissues were utilized as control samples. None of the patients included in the research had undergone radiation or chemotherapy. The clinical characteristics of the study participants are listed in **Table 1**. The experiment received approval from Guangzhou Medical University Hospital, with the approval number GD2019-036, in compliance with the principles outlined in the Helsinki Declaration (25). The evaluation and classification of the disease’s surgical staging were conducted based on the updated surgical staging criteria for endometrial cancer (EC) established by the International FIGO in 2009. A total of 28 instances of FIGO type III-IV and 14 instances of FIGO type I-II were gathered.

**Table 1:** Clinical characteristics of the study participants.

Variables	Number	Percent
Age (years)		
≤50	6	18.75%
>50	26	81.25%
Histology		
Type I	28	87.50%
Type II	4	12.50%
Surgical staging		
I-II	14	43.75%
III-IV	18	56.25%
Estrogen receptor status		
Negative	15	46.88%
Positive	17	53.13%
Progesterone receptor status		
Negative	14	43.75%
Positive	18	56.25%
Estrogen receptor and progesterone receptor status		
Single positive or negative	16	50.00%
Double positive	16	50.00%
Total	32	100.00%

### 2.2 Gene expression omnibus dataset analysis

Sample GSE25405, which was split into two groups—non-cancerous endometrial tissue and endometrioid adenocarcinoma tissue—produced differently expressed miRNAs. Sample GSE63678, which was split into two groups—healthy receiver tissue and EC patient tissue—produced the differentially expressed mRNAs. The miRNAs/mRNAs that satisfied the following criteria: *P* < 0.05; and log2|fold change (FC)| ≥ 2; they were deemed differentially expressed and may be used for pathway enrichment analysis, heatmap creation, volcano plotting, or Kyoto Encyclopedia of Genes and Genomes (KEGG) analysis. The binding sites of lncRNA/miRNA/mRNA were predicted using the lncBase v.3 and starBase databases. The GEO website provided the raw data (GSE25405 and GSE63678).

### 2.3 Cell culture and transfection

EC cell lines were maintained (37°C, 5% CO_2_) in DMEM (Gibco, Thermo Fisher Scientific, Inc.) supplemented with 10% fetal bovine serum (FBS; Gibco), included HEC-1-A, HEC-1-B, RL95-2, Ishikawa, AN3 CA (American Type Culture Collection), JEC (YRGene), and human endometrial epithelial cells (hEECs; Procell, Wuhan, China). Using Lipofectamine^®^ 2000 (Invitrogen, Thermo Fisher Scientific, Inc.), target cells were transfected with miR-140-3p mimic/inhibitor, small interfering (si) RNA JPX, or overexpression (ov) PIK3CA plasmid, along with their corresponding negative controls (NC), for a duration of 4 hours. **Table 2** shows the sequences for the siRNA-JPX, miR-140-3p mimic/inhibitor, and ov-PIK3CA that were manufactured by GenePharma Biotechnology Co, Ltd. (Shanghai, China).

**Table 2:** Sequences of the three siRNAs targeting JPX and miR-140-3p mimic or inhibitor.

siRNAs	Forward oligonucleotide 5′-3′	Reverse oligonucleotide 5′-3′
si-JPX-1	GAUUAUCUGUUUGAGUUAAAG	UUAACUCAAACAGAUAAUCAG
si-JPX-2	GUGACUUUCCAGUCAUUAAGA	UUAAUGACUGGAAAGUCACGG
si-JPX-3	GAGUUAAAGACAACAUCAUGG	AUGAUGUUGUCUUUAACUCAA
si-NC	UUCUCCGAACGUGUCACGUTT	ACGUGACACGUUCGGAGAATT
Sequences of miR-140-3p mimic or inhibitor 5′-3′		
Mimic NC	UAAGCGGAGACCACCAGAGCU	
Mimic	UACCACAGGGUAGAACCACGG	
Inhibitor NC	CAGUACUUUUGUGUAGUACAA	
Inhibitor	CCGTGGTTCTACCCTGTGGTA	

### 2.4 Subcellular localization of the lncRNA JPX

Centrifugation was used to gather the cells after two PBS washes. The manufacturer’s instructions for the nuclear and cytoplasmic protein extraction kit (Beyotime, Shanghai, China) directed resuspending 200 μL with an RNase inhibitor. Reagents containing PMSF were added after 15 minutes, and the mixture was centrifuged (16,000 × *g*; 10 minutes; 4°C). The supernatant was then collected for RT-qPCR analysis and total RNA extraction.

### 2.5 RNA pull-down assay

Biotinylated JPX (Bio-JPX) and the corresponding negative control (Bio-miR-NC) were synthesized by GenePharma. Cells were lysed, and the lysates incubated with the biotinylated RNA probes. Streptavidin-coated magnetic beads (Invitrogen) were added to capture the biotinylated RNA complexes. The pulled-down RNA was extracted and analyzed by RT-qPCR to determine the enrichment of miR-140-3p.

### 2.6 RT-qPCR assays

TRIzol reagent was used to extract total RNA from target cells. PrimeScript RT Kit (Takara Bio, Japan) was used to reverse transcribe 10 μL of DEPC water (Invitrogen) into cDNA from the precipitate after centrifugation at 12,000 × *g* (4°C; 10 min) and supernatant was adsorbed and disposed of. With an Applied Biosystems^®^ 7500 Real-Time PCR (California, USA) and the Takara SYBR^®^ Premix Ex TaqTM II kit, RT-qPCR analysis was carried out. The following temperatures were used for PCR experiments: 95°C for 10 minutes, 55°C for 2 minutes, and 72°C for 2 minutes. These were followed by 40 cycles of 95°C for 15 s and 60°C for 32 s. The sequences of the primer pairs are listed in **Table 3**. The housekeeping genes GAPDH and U6 served as a reference for normalizing the target RNA levels. The 2^−ΔΔCt^ method (26), which calculates relative RNA levels, was used.

**Table 3:** Primer sequences used in this study.

Primers	Forward primer 5′-3′	Reverse primer 5′-3′
JPX	GGCGTCCGAAGTATGAGTCC	TGCAACTTCCAAGCTTCGTC
miR-140-3p	ACACTCCAGCTGGGTACCACAGGGTAGAA	CTCAACTGGTGTCGTGGA
U6	CTCGCTTCGGCAGCACA	AACGCTTCACGAATTTGCGT
*PIK3CA*	TTACCCTCTTCTGCCGGAGG	TGTCCCAAAGCAGAAACATCGT
*GAPDH*	GCTCATTTGCAGGGGGGAG	GTTGGTGGTGCAGGAGGCA

U6 was used to normalize miR-140-3p expression, and GAPDH was used to normalize JPX and PIK3CA expression.

### 2.7 Dual luciferase assays

MiR-140-3p mimic, inhibitor, plasmids expressing wild-type (WT) or mutant (mut) JPX or PIK3CA, and pRL-SV40 reporter vector plasmid were used to transfect Ishikawa and JEC cells using Lipofectamine^®^ 2000. After 48 hours of incubation, the Dual-Luciferase Reporter Assay System (Promega) was used to assess the amount of luciferase activity in the transfected cells at 490 nm. The firefly luciferase data were normalized using the ratio of firefly to *Renilla* luciferase activity.

### 2.8 Western blotting

RIPA lysis buffer was used to lyse Ishikawa and JEC cells. A BCA protein assay kit (Solarbio) was used to estimate the protein contents in the lysate. Proteins that had been denatured were separated using 10% SDS-PAGE (Solarbio). The separated protein bands were then placed onto a PVDF membrane, blocked using 5% bovine serum albumin (Solarbio), and left to incubate at 4°C for an entire night using primary antibodies form Abcam (Cambridge, UK) against PIK3CA (1:2000; ab40776), JAK2 (1:3000; ab108596), p-JAK2 (1:1000; ab32101), STAT3 (1:1000; ab68153), and p-STAT3 (1:1000; ab267373). The sections were then treated with goat anti-rabbit antibody (1:5000, ab205718) for 2 hours at 25°C after being washed twice with TBST buffer (Solarbio) for 15 minutes. As a loading control, anti-GAPDH antibody (1:3000, ab181602) was used. The bound proteins were visualized by enhanced chemiluminescence (Thermo Fisher Scientific, Inc.). Digital images of protein bands were captured using an imaging system (Bio-Rad, USA) and bands were quantified using ImageJ software (National Institutes of Health, USA).

### 2.9 Cell viability and colony formation assay

Cell Counting Kit-8 (CCK-8) reagent (Solarbio; 10 μl) was applied at 0 and 72 hours to 96-well plates containing JEC 4 × 10^3^ and Ishikawa cells. After 60 minutes of dark incubation at 20°C, absorbance was measured at 450 nm with enzyme-labeled instrument (Thermo Fisher Scientific). Transfected cells were seeded onto 6-well plates for the colony formation test, and the plates were then incubated for two weeks. After being stained with 0.1% crystal violet (Solarbio) and treated with 4% paraformaldehyde, colonies were manually counted.

### 2.10 Apoptosis assay

For flow cytometry (FCM), Ishikawa and JEC cells were incubated (15 min) with Annexin V-FITC, followed by the addition of propidium iodide (PI) for another 5 min. FCM (BD FACSCalibur, BD Biosciences, CA, USA) was used to analyze cell apoptosis. Incubation was performed in the dark at 4°C. For acridine orange (AO)/ethidium bromide (EB) staining, cells were stained (20 min) with a mixture of AO and EB (Sigma-Aldrich, MO, USA), visualized under a fluorescence microscope (Olympus, Tokyo, Japan).

### 2.11 Cell cycle assay

Ishikawa and JEC cell suspensions were centrifuged (1,000 × *g*, 5 min) and immobilized in 70% ethyl alcohol at 4°C overnight. After washing with PBS, the cells were resuspended in PBS containing PI and RNase A (containing 0.2% Triton X-100). After 30 min of incubation at 4°C in the dark, FCM was used to analyze the cell cycle.

### 2.12 Transwell assay

For the invasion assay, Matrigel^®^ coating (BD Biosciences) was first applied at 37°C for 6 h. Ishikawa and JEC cells (1 × 10^5^) were placed in the upper chamber (serum-free medium), and 10% FBS was added to the lower chamber. After 48 h of incubation, the upper chamber was gently rinsed twice with PBS and then fixed with absolute ethanol for 20 min. After natural air drying, cells were stained (20°C, 15 min) with crystal violet. In migration detection the Matrigel coating is omitted. The cells were counted under a light microscope (Olympus Corporation; magnification, 200×).

### 2.13 Statistical analysis

Data are presented as mean ± standard deviation. Statistical analyses were performed with GraphPad Prism software. Student’s t-test or one-way ANOVA followed by Tukey’s *post hoc* test was used to compare groups. Pearson correlation analysis was used to analyze relationships between gene expression levels. Differences were considered significant at *P* < 0.05.

## 3 Results

### 3.1 Upregulated expression of JPX in EC

**Figure 1:**
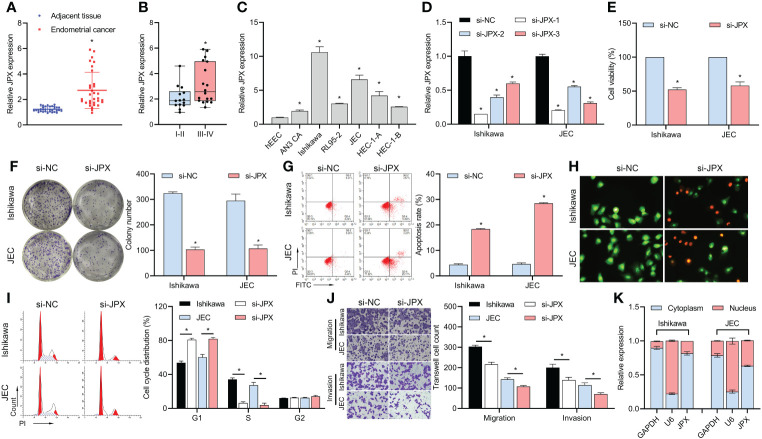
JPX expression and its functional role in EC cells. **(A)** RT-qPCR analysis of JPX expression in EC tissues and adjacent non-cancerous tissues. **(B)** Box plot showing association of JPX expression with EC prognosis. **(C)** RT-qPCR analysis of JPX expression in EC cell lines. **(D)** RT-qPCR analysis of JPX knockdown efficiency using si-JPXs in Ishikawa and JEC cells. **(E)** CCK8 analysis of the effects of JPX knockdown on cell viability in Ishikawa and JEC cells. **(F)** Colony formation assay assessing the impact of JPX knockdown on colony formation in Ishikawa and JEC cells. **(G, H)** Apoptosis assays (FCM and AO/EB) evaluating the influence of JPX knockdown on cell apoptosis in Ishikawa and JEC cells. **(I)** Cell cycle analysis of the effect of JPX knockdown on cell cycle progression in Ishikawa and JEC cells. **(J)** Transwell assay assessing the role of JPX knockdown on cell migration and invasion in Ishikawa and JEC cells. **(K)** Nuclear-cytoplasmic fractionation experiments examining the subcellular localization of lncRNA JPX. *P<0.05.

According to our findings, EC tissues had higher levels of lncRNA JPX expression than adjacent non-cancerous tissues (**Figure 1A**). Moreover, there was a correlation found between the expression of JPX and the prognosis of EC, with higher levels in advanced stage EC compared to early stage EC (**Figure 1B**). When compared to hEEC cells, JPX expression was elevated *in vitro* in six EC cell lines (**Figure 1C**), with Ishikawa and JEC cells exhibiting the highest levels. To better understand the molecular processes of JPX in EC cells, we have chosen these two cell lines. After constructing three siRNAs targeting JPX, we observed a significant downregulation of JPX expression in Ishikawa and JEC cells, especially with si-JPX-1, which showed the best inhibitory effect (**Figure 1D**). We selected si-JPX-1 as a JPX antagonist for further studies. We then found that inhibition of JPX expression reduced cell viability (**Figure 1E**) and colony formation (**Figure 1F**), promoted cell apoptosis (**Figures 1G, H**), and induced cell cycle arrest (**Figure 1I**), while decreasing cell migration and invasion abilities in Ishikawa and JEC cells (**Figure 1J**). Notably, lncRNA JPX was mainly expressed in the cytoplasm, consistent with the positive control GAPDH and opposite to the positive control U6 (**Figure 1K**).

### 3.2 miR-140-3p is a direct target of lncRNA JPX

**Figure 2:**
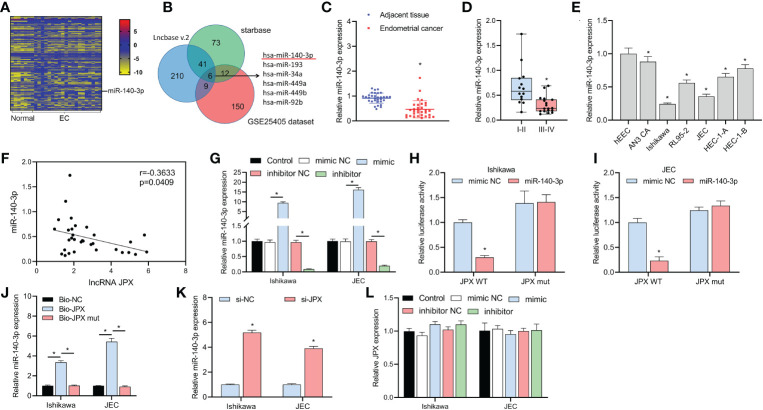
Identification of miR-140-3p as a direct target of lncRNA JPX. **(A)** GEO data analysis of differentially expressed miRNAs in EC tissues. **(B)** Venn diagram showing the joint analysis using Lncbase v.2 and starBase databases to identify hypothetical miRNA targets of JPX. **(C)** RT-qPCR analysis of miR-140-3p expression in EC tissues. **(D)** Box plot showing association of miR-140-3p expression with EC prognosis. **(E)** RT-qPCR analysis of miR-140-3p expression in EC cell lines. **(F)** Pearson correlation analysis between the expression of miR-140-3p and lncRNA JPX. **(G)** RT-qPCR analysis of miR-140-3p mimic and inhibitor efficiency in EC cells. **(H, I)** Dual-luciferase assays analyzing the interaction between JPX and miR-140-3p in Ishikawa **(H)** and JEC **(I)** cell lines. **(J)** RNA pull-down assay results showing miR-140-3p enrichment in the Bio-JPX group. **(K)** RT-qPCR analysis of miR-140-3p expression following JPX inhibition. **(L)** RT-qPCR analysis of JPX expression after miR-140-3p manipulation. *P<0.05.

We discovered 150 differentially expressed miRNAs by examining the GSE25405, a dataset (**Figure 2A**). Six putative target miRNAs of JPX were identified by combined analysis utilizing the Lncbase v.2 and starBase databases (**Figure 2B**): miR-140-3p, miR-193a-3p, miR-34a-5p, miR-449a, miR-449b-5p, and miR-92b-3p. Moreover, miR-140-3p was the only miRNA that was downregulated in EC tissues among these six miRNAs. MiR-140-3p expression was shown to be downregulated in cancer tissues (**Figure 2C**) and its expression level was linked to the prognosis of EC, with lower expression in advanced stage EC compared to early stage EC (**Figure 2D**). These findings were obtained by further RT-qPCR research. Compared to hEEC cells, six EC cell lines had lower miR-140-3p expression *in vitro* (**Figure 2E**). The levels of miR-140-3p and lncRNA JPX showed a negative linear connection, according to Pearson correlation analysis (**Figure 2F**). The connection between JPX and miR-140-3p was examined using dual-luciferase assays after the validation of miR-140-3p mimics and inhibitors (**Figure 2G**). In comparison to the WT-JPX + mimic NC group, the luciferase activity was lower in the co-transfection group of WT-JPX + mimic, according to the findings of the dual-luciferase evaluation. Furthermore, no discernible variations in luciferase activity were seen between the mut-JPX and mimic NC or mimic co-transfection groups (**Figures 2H, I**). Following site modification, RNA pull-down analysis revealed miR-140-3p enrichment in the bio-JPX group, with no discernible alterations when compared to the bio-miR-NC group (**Figure 2J**). Moreover, JPX expression inhibition increased the expression of miR-140-3p (**Figure 2K**), however JPX was not substantially affected by changes in miR-140-3p expression (**Figure 2L**).

### 3.3 Interaction between JPX and miR-140-3p

**Figure 3:**
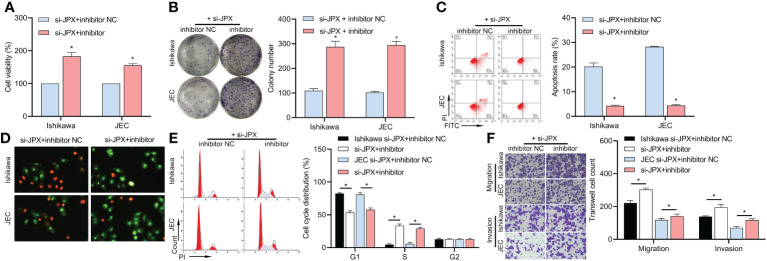
Interaction between JPX and miR-140-3p in EC cells. **(A)** CCK8 analysis of the effects of miR-140-3p inhibitor on cell viability in JPX-silenced Ishikawa and JEC cells. **(B)** Colony formation assay assessing the impact of miR-140-3p inhibitor on colony formation in JPX-silenced Ishikawa and JEC cells. **(C, D)** Apoptosis assays (FCM and AO/EB) evaluating the influence of miR-140-3p inhibitor on cell apoptosis in JPX-silenced Ishikawa and JEC cells. **(E)** Cell cycle analysis of the effect of miR-140-3p inhibitor on cell cycle progression in JPX-silenced Ishikawa and JEC cells. **(F)** Transwell assay assessing the role of miR-140-3p inhibitor on cell migration and invasion in JPX-silenced Ishikawa and JEC cells. *P<0.05.

We co-transfected inhibitor NC or inhibitor with si-JPX. The decrease of Ishikawa and JEC cell viability (**Figure 3A**), colony formation (**Figure 3B**), apoptosis (**Figures 3C, D**), cell cycle arrest (**Figure 3E**), and cell migration and invasion (**Figure 3F**) was reversed by the miR inhibitor, according to the results.

### 3.4 miR-140-3p directly targets PIK3CA

**Figure 4:**
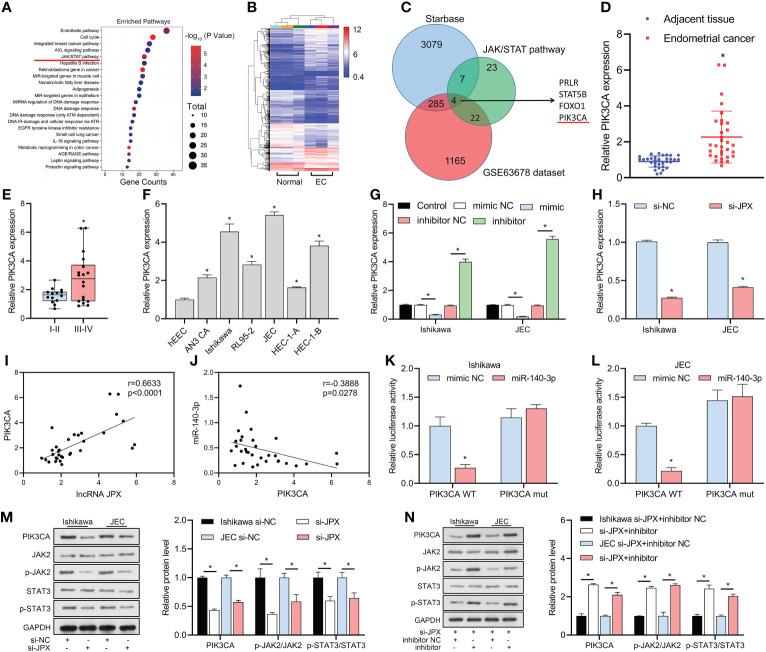
miR-140-3p directly targets PIK3CA and its association with the JAK/STAT pathway. **(A)** KEGG analysis revealing the JAK/STAT pathway as a critical pathway affecting EC progression. **(B)** GEO data analysis of differentially expressed mRNAs in EC tissue. **(C)** Venn diagram showing the identification of potential target mRNAs of miR-140-3p affecting the JAK/STAT pathway. **(D)** RT-qPCR analysis of PIK3CA expression in EC tissues. **(E)** Box plot showing association of PIK3CA expression with EC prognosis. **(F)** RT-qPCR analysis of PIK3CA expression in EC cell lines. **(G)** RT-qPCR analysis of PIK3CA expression following miR-140-3p manipulation. **(H)** RT-qPCR analysis of PIK3CA expression after JPX inhibition. **(I)** Pearson correlation analysis between PIK3CA expression and lncRNA JPX. **(J)** Pearson correlation analysis between PIK3CA expression and miR-140-3p. **(K, L)** Dual-luciferase assay results showing the interaction between PIK3CA and miR-140-3p in Ishikawa **(K)** and JEC **(L)** cell lines. **(M)** Western blot analysis of PIK3CA protein levels and JAK2/STAT3 phosphorylation following JPX knockdown. **(N)** Western blot analysis of PIK3CA protein levels and JAK2/STAT3 phosphorylation after miR-140-3p inhibitor treatment in JPX-silenced cells. *P<0.05.

KEGG analysis revealed that the JAK/STAT pathway might be a critical pathway in EC (**Figure 4A**) and identified 23 mRNAs affecting the JAK/STAT pathway. The GSE63678 data set revealed 1165 differentially expressed mRNAs in EC tissues compared to the normal group (**Figure 4B**). We analyzed the hypothetical target of miR-140-3p using the starBase database and combined them with the 23 mRNAs affecting the JAK/STAT pathway to narrow the scope of the mRNA screening. The results predicted that four mRNAs (PRLR, STAT5B, FOXO1, PIK3CA) were potential targets (**Figure 4C**). Among them, GEO data analysis showed that PIK3CA was the only up-regulated mRNA, with increased expression in cancer tissues (**Figure 4D**) and higher expression in advanced stage EC than in early stage EC (**Figure 4E**). *In vitro*, PIK3CA expression was upregulated in six EC cell lines compared to hEEC cells (**Figure 4F**). PIK3CA expression was negatively regulated by miR-140-3p (**Figure 4G**) and decreased when JPX expression was inhibited (**Figure 4H**). PIK3CA expression and lncRNA JPX had a positive linear connection, according to Pearson correlation analysis (**Figure 4I**); miR-140-3p and PIK3CA expression had a negative linear association (**Figure 4J**). The luciferase activity was decreased in the WT-PIK3CA + mimic co-transfection group compared to the WT-PIK3CA + NC mimic group. In addition, no differences in luciferase activity between the mut-PIK3CA and either the NC or mimic co-transfection groups (**Figures 4K, L**). Furthermore, suppression of JPX expression in Ishikawa and JEC cells resulted in decreased PIK3CA protein levels and dephosphorylation of JAK2/STAT3 (**Figure 4M**). Under the influence of miR-140-3p inhibitor, this effect was reversed (**Figure 4N**). Thus, PIK3CA may be a critical factor in the lncRNA JPX/miR-140-3p-mediated regulation of the JAK2/STAT3 pathway.

### 3.5 Interaction between JPX and PIK3CA

**Figure 5:**
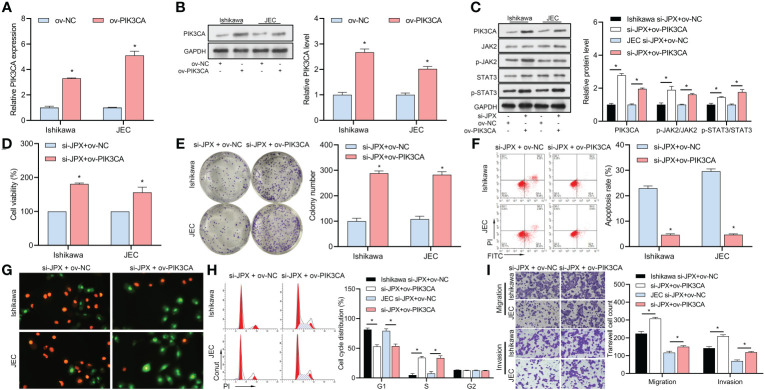
Interaction between JPX and PIK3CA in EC cells. **(A, B)** Validation of PIK3CA overexpression vector efficiency using RT-qPCR **(A)** and western blot **(B)** in Ishikawa and JEC cell lines. **(C)** Western blot analysis of PIK3CA protein levels and JAK2/STAT3 phosphorylation after PIK3CA overexpression in JPX-silenced cells. **(D)** CCK8 analysis of the effects of PIK3CA overexpression on cell viability in JPX-silenced Ishikawa and JEC cells. **(E)** Colony formation assay assessing the impact of PIK3CA overexpression on colony formation in JPX-silenced Ishikawa and JEC cells. **(F, G)** Apoptosis assays (FCM and AO/EB) evaluating the influence of PIK3CA overexpression on cell apoptosis in JPX-silenced Ishikawa and JEC cells. **(H)** Cell cycle analysis of the effect of PIK3CA overexpression on cell cycle progression in JPX-silenced Ishikawa and JEC cells. **(I)** Transwell assay assessing the role of PIK3CA overexpression on cell migration and invasion in JPX-silenced Ishikawa and JEC cells. *P<0.05.

After validating the efficacy of the PIK3CA overexpression vector (**Figures 5A, B**), we found that it reversed the si-JPX-induced reduction of PIK3CA protein levels and dephosphorylation of JAK2/STAT3 (**Figure 5C**). Subsequently, we observed that si-JPX-mediated reduction of Ishikawa and JEC cell viability (**Figure 5D**) and colony formation (**Figure 5E**), increased cell apoptosis (**Figures 5F, G**), cell cycle arrest (**Figure 5H**), and decreased cell migration and invasion abilities (**Figure 5I**) were all reversed by PIK3CA overexpression.

## 4 Discussion

Progesterone treatment in the early stages of EC has a better therapeutic effect in improving the survival rate and reducing the risk of treatment. Patients with intermediate and advanced stages undergo surgery or chemotherapy combined with drugs such as gefitinib (27) and cisplatin (28); however, the prognosis is still poor (29). Therefore, the study of its pathological mechanism is urgent. As markers of EC, lncRNAs have attracted much attention in recent years (30). Research has shown that the abnormal expression of lncRNAs (such as TDRG1 (31) and NEAT1 (32)) is associated to the development of EC. A study by Chen et al. (7) in 2020 demonstrated that lncRNA JPX promotes the progression of cervical cancer; therefore, JPX may also be involved in the progression of EC. We demonstrated that lncRNA JPX was upregulated in EC tissues, and associated with the prognosis of EC, with higher expression in advanced-stage EC than early-stage EC. Furthermore, JPX is highly expressed in EC cell lines, which is similar to the findings of Chen et al. (7). After suppressing the expression of JPX, the viability, colony formation, and migration ability of Ishikawa and JEC cells was inhibited, and cell apoptosis and cycle arrest were enhanced, suggest that suppression of JPX expression hinders the progression of EC. Therefore, lncRNA JPX act as a novel target for the treatment of EC.

Due to the mechanism of ceRNA, we jointly screened JPX’s target miRNA through GEO analysis and the starBase database. We found that miR-140-3p may be the best choice. Subsequently, this study confirmed for the first time that JPX acts as a natural sponge to adsorb miR-140-3p through dual luciferase and RNA pull-down experiments. The reason for screening miR-140-3p is that it is downregulated in EC, and lncRNA JPX promotes the development of EC by adsorbing miR-140-3p. In addition, continuous inhibition of miR-140-3p can reverse the inhibition of the low level of JPX in the development of EC. Increasing miR-140-3p transcription and targeting PIK3CA inhibition may be the key to controlling the development of EC.

MiRNAs can bind to the 3’UTR end of mRNA and inhibit the transcription and translation of mRNA (33). This study used the starBase database, KEGG, and GEO data mining to jointly screen the downstream targets of miR-140-3p. In addition, we found that PIK3CA may be a key participant in the process of EC, which has been confirmed by many studies (34, 35). The mutation of PIK3CA triggers the downstream signal cascade through JAK/STAT and participates in cell viability, differentiation, and apoptosis (36). Li et al. (37) found that PIK3CA is also a key factor in the JAK/STAT pathway, which is consistent with our KEGG analysis results. Subsequently, we confirmed for the first time that miR-140-3p targets PIK3CA and regulates the viability, apoptosis, cycle, and migration of Ishikawa and JEC cells through RIP, dual luciferase, and cell function experiments. Furthermore, the suppression of JPX expression induced the downregulation of PIK3CA protein levels and JAK2/STAT3 phosphorylation levels, and the presence of miR-140-3p inhibitor and ov-PIK3CA reversed the effects of si-JPX. These results suggest that JPX regulates PIK3CA/JAK2/STAT3 signaling through miR-140-3p and promotes the progression of EC.

This study, however, had several limitations. First, due to the small patient sample size, the clinical data of this study still lack universality. Second, we have not yet confirmed the effects of JPX in animals. Finally, GEO data show that there are still many information networks connected by the lncRNA-miRNA-mRNA signaling pathway, including the potential regulation of JPX on other downstream signals, which may considerably affect the treatment of EC. These are the directions for future research.

In conclusion, we demonstrated that JPX is upregulated in EC tissues and cell lines, and its high expression is associated with poor prognosis. By functioning as a ceRNA, JPX negatively regulates miR-140-3p, which in turn targets PIK3CA. This interaction leads to the activation of the JAK2/STAT3 signaling pathway, thereby promoting EC cell proliferation, migration, and invasion while inhibiting apoptosis and inducing cell cycle arrest. Our findings provide new insights into the molecular mechanisms underlying EC progression and suggest that lncRNA JPX could serve as a promising target for treatment.

## Data availability statement

Publicly available datasets were analyzed in this study. This data can be found here: [https://www.ncbi.nlm.nih.gov/geo/query/acc.cgi?acc=GSE25405 and https://www.ncbi.nlm.nih.gov/geo/query/acc.cgi?acc=GSE63678].

## Ethics statement

The studies involving humans were approved by The ethics committee of the Guangzhou Medical University Hospital. The studies were conducted in accordance with the local legislation and institutional requirements. The participants provided their written informed consent to participate in this study. Ethical approval was not required for the studies on animals in accordance with the local legislation and institutional requirements because only commercially available established cell lines were used. Written informed consent was obtained from the individual(s) for the publication of any potentially identifiable images or data included in this article.

## Author contributions

WZ: Conceptualization, Data curation, Formal analysis, Writing – review & editing. MX: Methodology, Software, Supervision, Validation, Visualization, Writing – original draft. RC: Formal analysis, Project administration, Resources, Supervision, Writing – original draft. HC: Conceptualization, Data curation, Formal analysis, Supervision, Writing – review & editing. HX: Conceptualization, Funding acquisition, Methodology, Resources, Validation, Visualization, Writing – original draft, Writing – review & editing. QL: Funding acquisition, Investigation, Resources, Writing – original draft, Writing – review & editing.
